# ZPTM: Zigzag Path Tracking Method for Agricultural Vehicles Using Point Cloud Representation

**DOI:** 10.3390/s25041110

**Published:** 2025-02-12

**Authors:** Shuang Yang, Engen Zhang, Yufei Liu, Juan Du, Xiang Yin

**Affiliations:** 1 School of Agricultural Engineering and Food Science, Shandong University of Technology, Zibo 255000, China; yangsh022@163.com (S.Y.); zeg_nice2mu@163.com (E.Z.); dujuan@sdut.edu.cn (J.D.); 2 College of Biosystems Engineering and Food Science, Zhejiang University, Hangzhou 310058, China; yufeiliu@zju.edu.cn

**Keywords:** agricultural vehicle, automatic navigation, point cloud, zigzag path tracking

## Abstract

Automatic navigation, as one of the modern technologies in farming automation, enables unmanned driving and operation of agricultural vehicles. In this research, the ZPTM (Zigzag Path Tracking Method) was proposed to reduce the complexity of path planning by using a point cloud consisting of a series of anchor points with spatial information, which are obtained from orthophotos taken by UAVs (Unmanned Aerial Vehicles) to represent the curved path in the zigzag. A local straight path was created by linking two adjacent anchor points, forming the local target path to be tracked, which simplified the navigation algorithm for zigzag path tracking. A nonlinear feedback function was established, using both lateral and heading errors as inputs for determining the desired heading angle of agricultural vehicles, which were guided along the local target path with minimal errors. A GUI (Graphic User Interface) was designed on the navigation terminal to visualize and monitor the working process of agricultural vehicles in automatic navigation, displaying interactive controls and components, including representations of the zigzag path and the agricultural vehicle using affine transformation. A high-clearance sprayer equipped with an automatic navigation system was utilized as the test platform to evaluate the proposed ZPTM. Zigzag navigation tests were conducted to explore the impact of path tracking parameters, including path curvature, moving speed, and spacing between anchor points, on zigzag navigation performance. Based on these tests, a regression model was established to optimize these parameters for achieving accurate and smooth movement. Field test results showed that the maximum error, average error, and RMS (Root Mean Square) error in the zigzag navigation were 3.30 cm, 2.04 cm, and 2.27 cm, respectively. These results indicate that the point cloud path-based ZPTM in this research demonstrates adequate stability, accuracy, and applicability in zigzag navigation.

## 1. Introduction

With the increasing application of cutting-edge technologies, precision agriculture has been experiencing significant development during the last decades [1,2]. Agricultural vehicles are becoming increasingly intelligent, with traditional agricultural vehicles gradually being replaced by smart ones [3,4]. Automatic navigation contributes as one of the core technologies in achieving precision agriculture, which has been widely used in agricultural vehicles for sowing, weeding, and harvesting to improve operation efficiency and reduce crop production costs [5,6,7].

The problem of automatic navigation for agricultural vehicles in complex field environments has received considerable research attention [8,9]. The purpose of automatic navigation is to control agricultural vehicles to travel accurately along predetermined paths and efficiently achieve full coverage of the operation area [10,11]. When the attitude information of agricultural machinery is accurately obtained through integrated navigation technology [12,13], the accuracy of agricultural vehicle automatic navigation is closely related to efficient path planning algorithms [14,15,16]. In order to deal with the challenges of path planning in complex environments, various path planning algorithms have been proposed. The A* algorithm effectively plans the optimal operation path for static operation scenarios with minimal cost, which has been widely used in the field of automatic navigation [17,18,19]. Jeon et al. [20] developed a novel path planner using the A* algorithm to generate an entry–exit path that enables the tractor to enter the farmland from the entrance, complete the agricultural task, and return. In two real paddy field tests, the entry–exit paths created by the proposed path planner effectively guided the tractor to reach the given target point with a lateral error of ≤ 8.5 cm. However, when the agricultural operation scene is large, and the navigation accuracy is high, the A* algorithm has the disadvantages of long search calculation times and high computer memory resources [21]. The pure tracking model is a geometric method to simulate the manual driving process, which uses the calculated arc to connect the vehicle body and the preview point [22,23,24]. A suitable local tracking path was planned by dynamically adjusting the forward-looking distance of the model according to the speed, lateral, and heading errors. Wu et al. [25] considered the impact of operating speed and target path curvature on the look-ahead distance of the agricultural vehicle, and the target path was planned by adjusting the look-ahead area and calculating the preview point. Curvature path tracking control experiments were conducted. The results showed that when the tractor was driving at speeds of 1.0 m/s, 1.5 m/s, 2.0 m/s, and 3.0 m/s, the average absolute errors were 2.7 cm, 2.7 cm, 3.3 cm, and 4 cm, respectively. He et al. [26] proposed a novel local adaptive tracking path planning method using pose error of agricultural vehicles by combining the pure tracking algorithm with fuzzy control. The straight-line path tracking tests in paddy fields were conducted to validate the stability and precision of the method. Compared with the traditional pure tracking algorithm, the proposed algorithm improves the performance of the straight-line path tracking of the tracked combine harvester. In recent years, meta-heuristic optimization algorithms have been widely applied to path planning due to their powerful global search capability and adaptability to deal with a wide range of constraints in complex farmland environments [27,28,29]. Khan et al. [30] proposed an RNN-based approach to the tracking control of redundant mobile manipulators, using a metaheuristic optimization process to find control parameters. This provides novel ideas for dealing with path planning in dynamic and complex farmlands.

In recent years, UAVs have been widely applied in various agricultural fields, including crop management [31,32], seed sowing, and pesticide spraying, due to their high efficiency and precision [33,34]. Wu et al. [35] proposed a navigation method that extracted the coordinate information of seedlings by utilizing UAVs to capture farmland images, recognizing the navigation path of seeding rows. The automatic navigation of the robot vehicle along the curved rice seedlings was realized in complex paddy field environments. Sun et al. [36] proposed an orchard path plan method based on UAV images to assist agricultural robots in pesticide spraying and fruit harvesting. For the automatic navigation of agricultural vehicles, path planning is required to cover both farmland and field roads, so that the agricultural vehicle can move along the field road to the entrance of the working area and return after completing its tasks in the fields. Therefore, the target path is usually complex and zigzag, rather than straight. The zigzag and changeable path should be reasonably planned to generate a smooth trajectory so that agricultural vehicles can track with minimum error. Therefore, we combine an efficient UAV with an image processing algorithm to plan the target path.

In this research, the ZPTM was proposed by using a point cloud to describe the target zigzag path. The point cloud contained anchor points with latitude and longitude, which were created from orthographic images obtained by an UAV and stored in turn as the navigation map. Zigzag path tracking was simplified by segment tracking between two adjacent anchor points. A nonlinear feedback function was established to determine the desired heading angle of agricultural vehicles using lateral and heading errors for highly precise path tracking. A GUI was developed to monitor the zigzag navigation process in real time and enable visualization of automatic navigation for agricultural vehicles. The path tracking parameters were optimized by establishing a regression model to explore their impact on zigzag navigation performance. Field tests were conducted to evaluate the stability and applicability of the proposed ZPTM.

## 2. Materials and Methods

**Figure 1:**
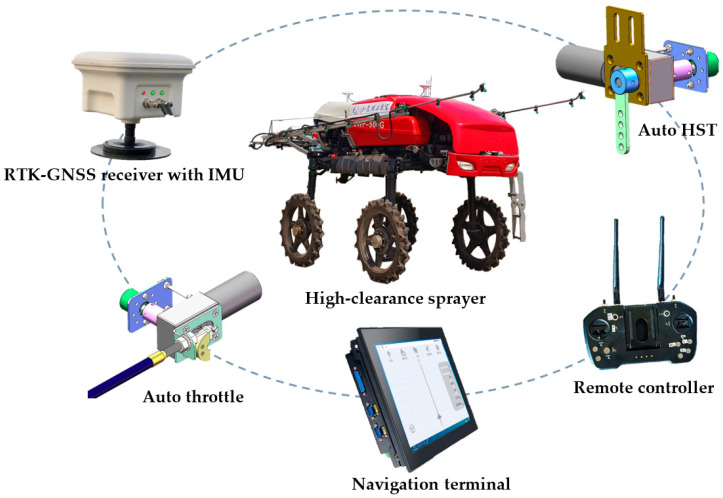
Components of test platform.

A high-clearance sprayer equipped with an electronic HST (Integrated Hydrostatic Transmission), automatic throttle, and automatic navigation with a CAN (Controller Area Network) bus communication system was utilized as the test platform, as shown in Figure 1 [37]. Its main parameters are described in Table 1. The automatic navigation system was developed by SDUT (Shandong University of Technology, Zibo, China) and is composed of an RTK-GNSS (Real-Time Kinematic Global Navigation Satellite System) receiver, a navigation terminal, a remote controller, and an IMU (Inertial Measurement Unit). Control commands, including steering and speed, were issued by the automatic navigation system, which automatically guided the high-clearance sprayer to travel along the target path. The RTK-GNSS receiver integrated a GNSS positioning board UM982 by Unicore Technology (Beijing) Co., Ltd., Beijing, China, to obtain centimeter-level positioning and heading information. The IMU (a JY61P model by Shenzhen Wit-Motion Intelligent Technology Co., Ltd., Shenzhen, China) was used to sense the vehicle posture. The navigation terminal, with interfaces including CAN and an RS232 serial port, was capable of receiving sensor data, running the navigation program, and providing controls for basic operations, including command output, navigation process monitoring, path planning, and map management. The terminal was configured with an Intel^®^ Core™ i5-13500H CPU @ 2.60 GHz and 16 GB RAM.

### 2.1. Path Planning

**Figure 2:**
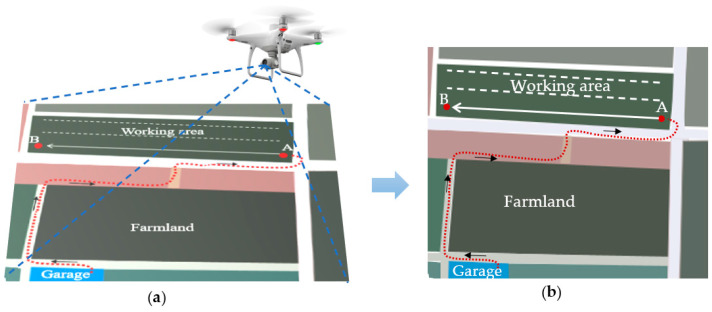
The process of (**a**) image acquisition by DJI Phantom 4 and (**b**) zigzag path planning.

A DJI Phantom 4 RTK UAV was used to capture a series of aerial images with a resolution of 5472 × 3648 pixels at a height of about 60 m, flying at a speed of 4.7 m/s over the SDUT ecological unmanned farm (118°13′ E, 36°57′ N), as shown in Figure 2a. The aerial images, containing farmland, a field road, and a garage, were stitched to form an orthophoto with geographic information for extracting the target zigzag path, as shown in Figure 2b. Anchor points were selected sequentially from the orthophoto to create a zigzag path, ensuring that agricultural vehicles could be guided to start from the garage, run along the field road to the working area, and return after completing assigned tasks. These anchor points were treated as a point cloud *Ω*, and their global positions were stored in a navigation map file.

(1) $$\Omega =\left\{\omega_{i}|\omega_{i}\in E^{3},1\leq i\leq N\right\},$$

where $\omega_{i}=(Lat,\;Lon,\;Code)$ contains the latitude, longitude, and operation information at each anchor point, and *N* is the number of anchor points in the point cloud.

**Figure 3:**
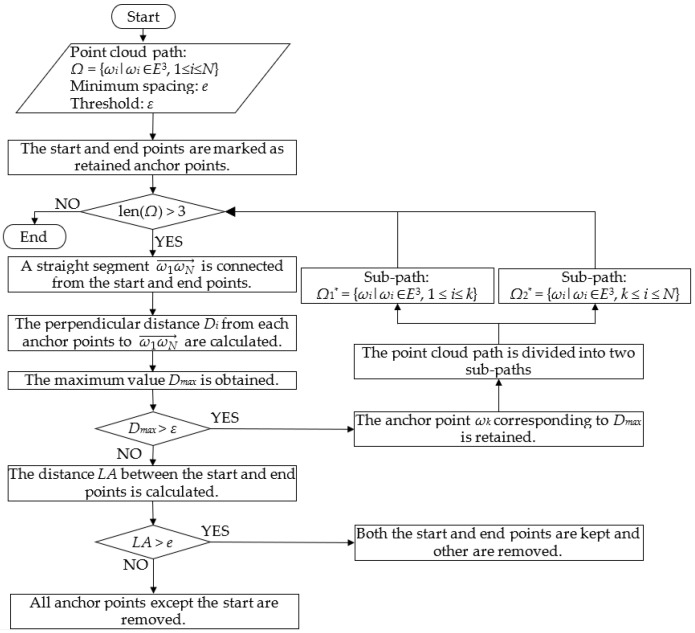
Flow chart of DPA.

The DPA (Douglas–Peucker Algorithm) [38,39] was utilized to remove redundant anchor points from the point cloud path to minimize the storage size of the map file while maintaining the original path shape, as shown in Figure 3. A minimum spacing e was given in the DPA to prevent over-simplification.

The original zigzag path consisted of multiple segments defined by connecting all anchor points in the point cloud *Ω*. The start and end points of the zigzag path were marked as retained anchor points, and a straight segment $\vec{\omega_{1}\omega_{N}}$ was drawn between them. The perpendicular distances *D_i_* from the other anchor points to this segment were calculated by Equations (2) and (3), and the maximum value *D_max_* was obtained.

(2) $$D_{i}=\frac{\vec{\omega_{i}^{\prime }\omega_{1}^{\prime }}\times \vec{\omega_{i}^{\prime }\omega_{N}^{\prime }}\cdot \vec{\omega_{i}^{\prime }\omega_{N}^{\prime }}}{\vec{\omega_{1}^{\prime }\omega_{N}^{\prime }}},2\leq i\leq N-1,$$

(3) $$\left\{\begin{array}{l} x_{i}=Rcos\left({Lat}_{i}\right)\cdot cos\left({Lon}_{i}\right)\\ y_{i}=Rcos\left({Lat}_{i}\right)\cdot sin\left({Lon}_{i}\right)\\ z_{i}=Rsin\left({Lat}_{i}\right) \end{array}\right.,$$

where *R* is the radius of the Earth, and $\omega_{i}^{\prime }=(x_{i},y_{i},z_{i})$ is the cartesian coordinate converted from the geographical coordinate $\omega_{i}=({Lat}_{i},{Lon}_{i})$.

In the case that *D_max_* was no more than the given threshold *ε*, all anchor points except the start point were considered to be insignificant and removed from the point cloud if the distance *LA* between the start and end points was no more than the minimum spacing *e*. If *LA* exceeded *e*, both the start and end points were kept, and the others were removed. In the case that *D_max_* exceeded *ε*, the anchor point $\omega_{k}$ corresponding to *D_max_* was kept, and the zigzag path was divided into two sub-paths that are represented by $\Omega_{1}^{*}$ and $\Omega_{2}^{*}$, which are defined in Equations (4) and (5), respectively. The sub-paths were processed iteratively using the DPA until there were no anchor points to be removed. In this way, a simplified zigzag path was obtained with fewer anchor points.

(4) $$\Omega_{1}^{*}=\left\{\omega_{i}^{*}|\omega_{i}^{*}\in E^{3},1\leq i\leq k\right\}$$

(5) $$\Omega_{2}^{*}=\left\{\omega_{i}^{*}|\omega_{i}^{*}\in E^{3},k\leq i\leq N\right\}$$

**Figure 4:**
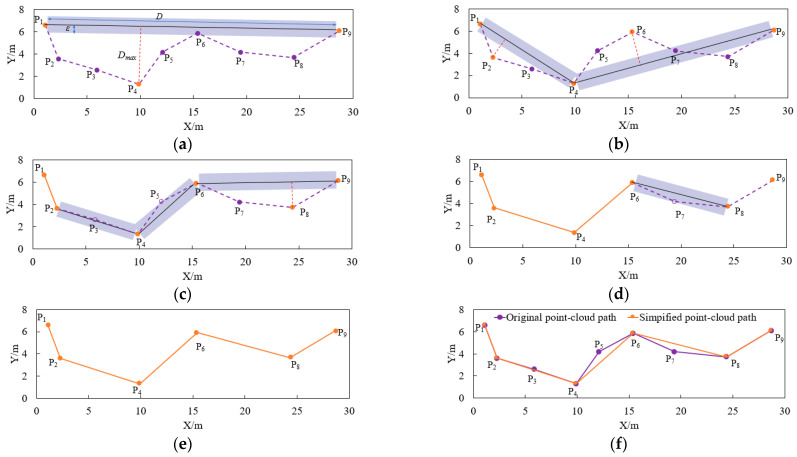
An example of the DPA: (**a**) Original point-cloud path. (**b**–**d**) Process using DPA. (**e**) Result after applying DPA. (**f**) Comparison of original and simplified point-cloud path.

As shown in Figure 4, the zigzag path consists of eight segments formed by connecting nine anchor points, which is simplified by applying the DPA with a threshold *ε* of 1 m and a minimum spacing *e* of 1 m. By comparing the simplified zigzag path with the original zigzag path, the RMSE (Root Mean Squared Error), MAE (Mean Absolute Error), and ME (Maximum Error) are found to be 0.34 m, 0.23 m, and 0.86 m, respectively. These results indicate that the DPA effectively simplifies the zigzag path by giving certain constraints.

### 2.2. Point Cloud Path Tracking

In this research, the ZPTM was developed to guide an agricultural vehicle along the point cloud path by minimizing both lateral and heading errors. The desired steering angle is determined by calculating the lateral error and heading error of the agricultural vehicle relative to the point cloud path in real time. The anchor points ω_i_^*^ = (*Lat*, *Lon*) near the vehicle is taken from the point cloud *Ω* to create a new subset Ω^*^, as defined in Equation (6).

(6) $$\Omega^{*}=\left\{\omega_{i}^{*}|\omega_{i}^{*}\in E^{2},1\leq i\leq N^{*}\right\}$$

**Figure 5:**
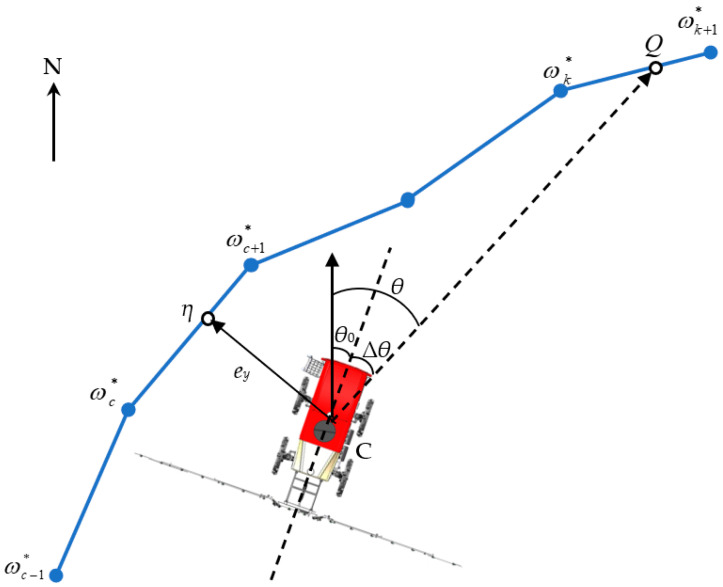
Point cloud path tracking algorithm: $\omega_{c-1}^{*},\;\omega_{c}^{*},\;\omega_{c+1}^{*},\;\omega_{k}^{*}$ and $\omega_{k+1}^{*}$ are anchor points in the point cloud path. C is the movement center of the agricultural vehicle chassis, representing the vehicle position. *η* is the nearest point from the vehicle position to the point cloud path. *Q* is the target point. *θ_0_* is the heading angle of the agricultural vehicle. *θ* is the desired heading angle of the agricultural vehicle. *∆θ* is the heading error defined as the angle between vector $\vec{CQ}$ and the heading of the vehicle. *e_y_* is the lateral error defined as the perpendicular distance from C to vector $\vec{\omega_{c}^{*}\omega_{c+1}^{*}}$.

The target zigzag path, determined by the subset Ω*, is shown in Figure 5. The position C of the agricultural vehicle is obtained from an RTK-GNSS receiver. The anchor point $\omega_{c}^{*}$ that is closest to position C of the agricultural vehicle is searched by using Equation (7).

(7) $$\omega_{c}^{*}=\left\{\omega_{c}^{*}|{argmin}_{i}{\left\|C-\omega_{c}^{*}\right\|}^{2},\omega_{c}^{*}\in \Omega^{*}\right\}$$

The local target path $\vec{\omega_{c}^{*}\omega_{c+1}^{*}}$ is constructed by connecting the nearest anchor point $\omega_{c}^{*}$ and the next anchor point $\omega_{c+1}^{*}$. The lateral error *e_y_* is defined as the perpendicular distance from C to $\vec{\omega_{c}^{*}\omega_{c+1}^{*}},$ as shown in Equation (8). *η* is the nearest point to the agricultural vehicle on the local target path.

(8) $$e_{y}=\frac{\left|\vec{\omega_{c}^{*}\omega_{c+1}^{*}}\times \vec{\omega_{c}^{*}C}\right|}{\left|\vec{\omega_{c}^{*}\omega_{c+1}^{*}}\right|}$$

The lookahead distance *L* is defined as the sum of the segments from the nearest point *η* to the target point *Q*. The target point *Q* is located between the anchor points $\omega_{k}^{*}$ and $\omega_{k+1}^{*}$ in the point cloud path. These anchor points are determined by Equation (9).

(9) $$\left\{\begin{array}{l} \left\|\vec{\eta \omega_{c+1}^{*}}\right\|+\sum_{i=c+2}^{k}\left\|\vec{\omega_{i}^{*}\omega_{i-1}^{*}}\right\|\leq L\\ \left\|\vec{\eta \omega_{c+1}^{*}}\right\|+\sum_{i=c+2}^{k}\left\|\vec{\omega_{i}^{*}\omega_{i-1}^{*}}\right\|\geq L \end{array}\right.$$

The target point *Q* is represented by anchor points $\omega_{k}^{*}$ and $\omega_{k+1}^{*},$ as shown in Equation (10).

(10) $$Q=\lambda \omega_{k}^{*}+\left(1-\lambda \right)\omega_{k+1}^{*},0\leq \lambda \leq 1$$

The desired heading angle *θ* is given by Equation (11).

(11) $$\theta ={cos}^{-1}\left(\frac{\vec{CQ}}{\left\|\vec{CQ}\right\|}\cdot \vec{n}\right),$$

where $\vec{n}$ is the unit vector oriented to the north direction in the UTM (Universal Transverse Mercator) coordinate framework.

The heading error *∆θ* is given by Equation (12).

(12) $$∆\theta =\theta -\theta_{0}$$

The desired steering angle *φ* is calculated by lateral error *e_y_* and heading error *∆θ*.

(13) $$\varphi =K_{1}e_{y}+K_{2}∆\theta$$

where *K*_1_ and *K*_2_ are proportional gains.

The values of *e_y_* and *Δθ* represent both the magnitude and direction of the deviation relative to the target zigzag path. When the agricultural vehicle is to the left of the target zigzag path, *e_y_* is negative, and when it is to the right, *e_y_* is positive. When the heading of the agricultural vehicle deviates to the left relative to the vector direction of the target zigzag path, *Δθ* is negative, and when it deviates to the right, *Δθ* is positive.

### 2.3. Design of the GUI

**Figure 6:**
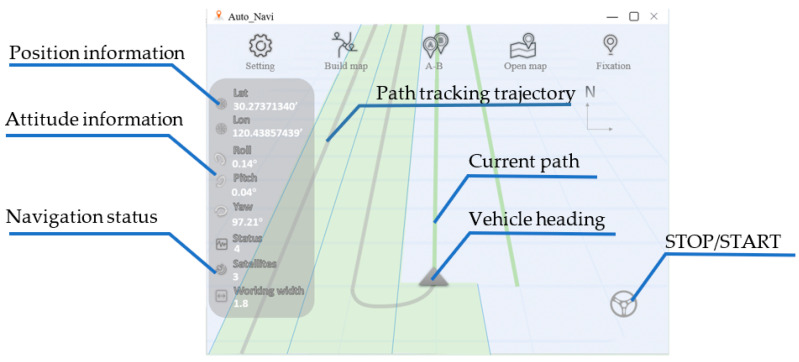
The GUI.

To visualize the working process, a GUI was developed on the navigation terminal, which served as an interactive medium between the user and the navigation system. Controls were designed to help the user in path planning, navigation map managing, safety monitoring, and information acquisition, as shown in Figure 6. Navigation data were displayed on the left, including attitude and position information of the agricultural vehicle, navigation status, and operation width. The STOP/START button on the bottom right was pressed to switch between the manual and automatic modes.

**Figure 7:**
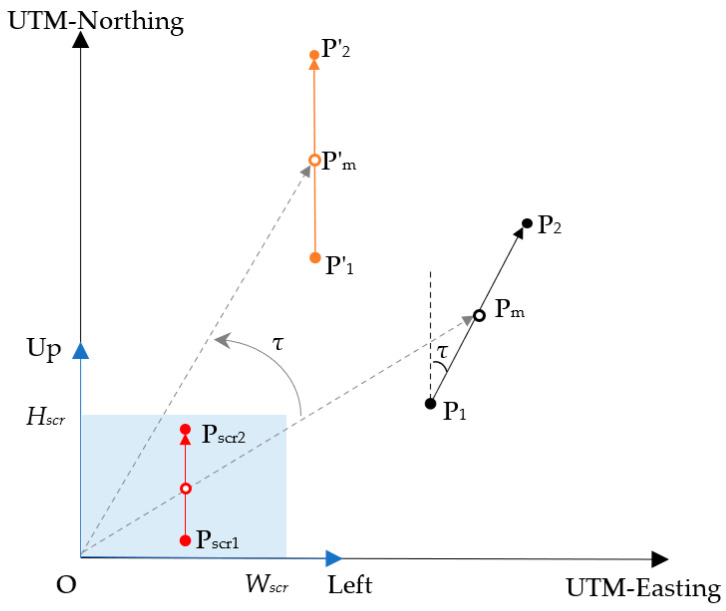
Coordinate transformation: Left-O-Up is the screen coordinate system. P_1_ and P_2_ are adjacent anchor points in the UTM coordinate system that form the local target path. P_m_ is the midpoint of the local target path. *τ* is the angle between the local target path and the UTM northing. P′_1_ and P′_2_ are the anchor points after rotation of P_1_ and P_2_, respectively. P_scr1_ and P_scr2_ are anchor points for converting the local target path to the screen. The screen resolution is *W_scr_* × *H_scr_*.

To monitor the working process on the navigation terminal, different elements were displayed on its screen to represent the agricultural vehicle and the target zigzag path. Since the position coordinates obtained from the RTK-GNSS receiver are in the geographic coordinate system, an affine transformation is necessary to convert these coordinates and orientations from the global frame, defined by WGS84 (World Geodetic System 1984) and UTM, to the screen frame. This affine transformation enables the visualization of the target zigzag path and the agricultural vehicle heading on the screen, aligning the local target path with the top of the screen, as shown in Figure 7.

Both the UTM coordinate system and the screen coordinate system utilize a common origin O for their coordinate frameworks. The local target path $\vec{P_{1}P_{2},}$ formed by adjacent anchor points, is rotated around the origin O by an angle *τ* so that its direction is the same as the UTM northing. Subsequently, in order to convert the rotated local target path $\vec{P_{1}^{\prime }P_{2}^{\prime }}$ from the UTM coordinate system to the screen coordinate system for full visualization, $\vec{P_{1}^{\prime }P_{2}^{\prime }}$ was scaled and translated with respect to the midpoint P′_m_ of $\vec{P_{1}^{\prime }P_{2}^{\prime }}$ so that the midpoint P′_m_ is located at the center of the screen. The scale factor *s* is determined based on the length of the local target path and the size of the screen, which is given by Equation (14). The translation vector (*t_x_*, *t_y_*) is calculated by Equation (15).

(14) $$s=\frac{H_{scr}}{\left\|\vec{P_{1}P_{2}}\right\|},$$

(15) $$\left\{\begin{array}{l} t_{x}=\frac{W_{scr}-s\left(x_{1}+x_{2}\right)}{2}\\ t_{y}=\frac{H_{scr}-s\left(y_{1}+y_{2}\right)}{2} \end{array}\right.,$$

where the (*x*_1_, *y*_1_) and (*x*_2_, *y*_2_) are coordinates of anchor point P_1_ and P_2_, respectively.

Finally, the anchor points P_1_ and P_2_ from the UTM coordinate system are converted to anchor points P_scr1_ and P_scr2_ in the screen coordinate system using the affine transformation, as shown in Equations (16) and (17).

(16) $$\left[\begin{array}{c} x_{scr1}\\ y_{scr1}\\ 1 \end{array}\right]=\left[\begin{array}{ccc} s\cdot cos\tau & -s\cdot sin\tau & t_{x}\\ s\cdot sin\tau & s\cdot cos\tau & t_{y}\\ 0 & 0 & 1 \end{array}\right]\left[\begin{array}{c} x_{1}\\ y_{1}\\ 1 \end{array}\right],$$

(17) $$\left[\begin{array}{c} x_{scr2}\\ y_{scr2}\\ 1 \end{array}\right]=\left[\begin{array}{ccc} s\cdot cos\tau & -s\cdot sin\tau & t_{x}\\ s\cdot sin\tau & s\cdot cos\tau & t_{y}\\ 0 & 0 & 1 \end{array}\right]\left[\begin{array}{c} x_{2}\\ y_{2}\\ 1 \end{array}\right],$$

where (*x_scr_*_1_, *y_scr_*_1_) and (*x_scr_*_2_, *y_scr_*_2_) are the coordinates of P_scr1_ and P_scr2_ in the screen coordinate system.

### 2.4. Optimization of Zigzag Path Tracking Parameters

Compared with straight tracking, the navigation performance of zigzag paths was affected greatly by three path tracking parameters, including the path curvature *φ*, the moving speed *v*, and the spacing *d* between anchor points. Therefore, the RMS value of lateral errors between the actual trajectory and the target zigzag path was selected to explore the influence of *φ*, *v,* and *d* on the navigation performance, which is calculated by Equation (18).

(18) $$e_{RMS}=\sqrt{\frac{\sum_{i=1}^{N}e_{i}^{2}}{N}},$$

where *e_i_* is the lateral error at the anchor point *ω_i_*.

**Figure 8:**
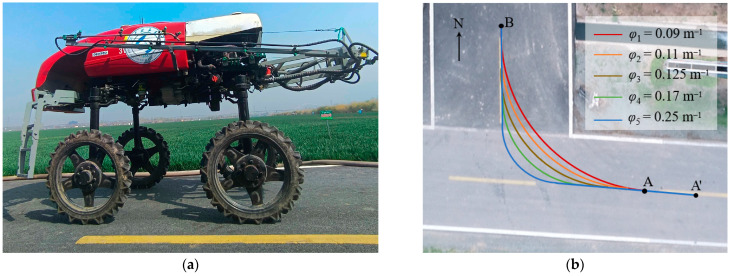
Zigzag navigation tests with (**a**) the high-clearance sprayer in (**b**) different zigzag path curvatures for the same turn.

Zigzag navigation tests were conducted for the same road at the ecological unmanned farm of SDUT using a high-clearance sprayer integrated with an automatic navigation system, as shown in Figure 8. During the test process, the values of three path tracking parameters were adjusted to investigate their relationship with lateral error, aiming to optimize the accuracy of zigzag navigation. Zigzag navigation maps were constructed by assigning diverse parameter values of curvature and spacing of anchor points. Considering the field road width and the minimum turning radius of the agricultural vehicle, the values of *φ* were set to 0.09 m^−1^, 0.11 m^−1^, 0.125 m^−1^, 0.17 m^−1,^ and 0.25 m^−1^. The values of *d* were set to 1.0 m, 1.5 m, 2.0 m, 2.5 m, and 3.0 m. The high-clearance sprayer tracked the target zigzag path based on the proposed ZPTM and fed back the real-time position through the RTK-GNSS receiver. The high-clearance sprayer started from A′ and drove along the zigzag path AB at a constant speed, completing the turn at point B. The values of *v* were set to 3.0 km/h, 4.0 km/h, 5.5 km/h, 7 km/h, and 8 km/h, respectively. The navigation terminal logged the actual trajectories of the high-clearance sprayer, with the operator manually recording lateral errors at each anchor point.

## 3. Results and Discussion

### 3.1. Determination of Optimal Navigation Parameters

**Figure 9:**
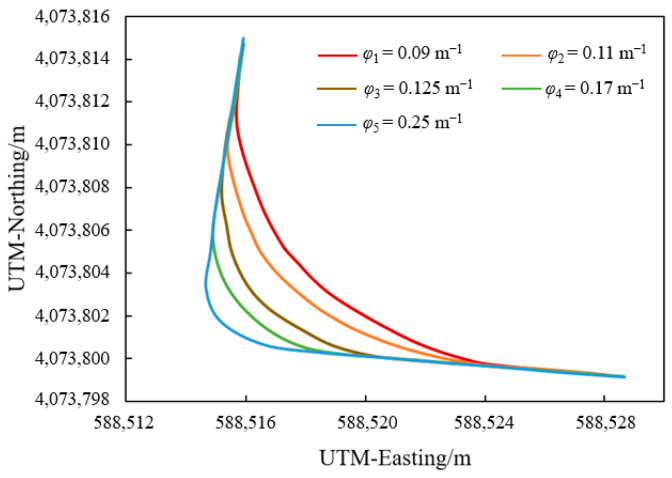
The actual trajectories of the high-clearance sprayer following five target zigzag paths.

The actual trajectories following the five target zigzag paths from point A to B are shown in Figure 9. The high-clearance sprayer started from point A′ (588,528.67, 4,073,799.147) and moved along the target zigzag path AB toward B at a constant speed. The turn was executed precisely when reaching A and finished at B. From Figure 9, it can be seen that the actual trajectory of the high-clearance sprayer on these five target zigzag paths is generally smooth. Lateral errors from 28 sets of zigzag navigation tests under different navigation parameters were recorded and analyzed, as shown in Table 2. However, relatively larger lateral errors occurred for the zigzag paths with curvature of 0.25 m^−1^, mainly due to its proximity to the minimum turning radius of the high-clearance sprayer. During the turning process, the wheels need to be rotated to their limit position, making it difficult for the agricultural vehicle to quickly and accurately track the target zigzag path. For those five target zigzag paths with different curvatures from A to B, the minimum, maximum, minimum average, maximum average, minimum RMS, and maximum RMS values were 1.6 cm, 6.8 cm, 0.75 cm, 2.69 cm, 0.85 cm, and 2.72 cm, respectively. This indicated that the proposed ZPTM had good tracking accuracy to meet the requirements for zigzag paths with different curvatures.

**Figure 10:**
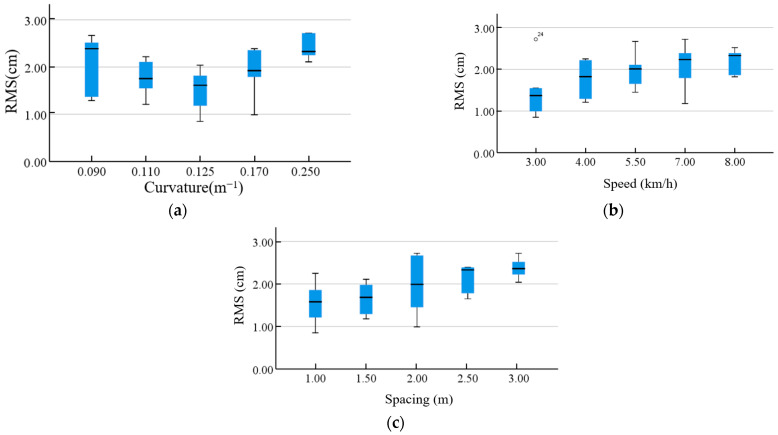
Comparative analysis of RMS in response to changes in (**a**) curvature, (**b**) speed, and (**c**) spacing.

Figure 10 shows a comparison between the RMS values for lateral errors obtained when the three path tracking parameters were changed. The RMS values significantly increased with the increase in the spacing of the anchor points and were proportional to the moving speed. Both excessively small and large curvature values resulted in increased RMS values. At a curvature of 0.125 m^−1^, the minimum RMS value reached 0.85 cm. The most significant variation in RMS values occurred with an anchor point spacing of 2 m, where errors ranged from 2.72 cm to 0.99 cm.

To obtain more robust analysis results, the ANOVA (Analysis of Variance) was conducted on the test results, and a regression model was established. The ANOVA results, after eliminating insignificant factors, are shown in Table 3. The results indicated that the regression model was statistically significant (F-value = 8.06, *p*-value < 0.05), and the effects of the curvature, moving speed, and the spacing of the anchor points on the lateral error of the zigzag navigation were statistically significant (*p*-value < 0.05). The regression model for the RMS value y was obtained as shown in Equation (19):

(19) $$y=0.170029+0.258064v+0.357539d-1.51609\varphi v+34.72536\varphi^{2}$$

The ANOVA results showed that spacing had the greatest impact on tracking accuracy based on the F-value. Path tracking parameters were optimized to minimize tracking errors. Based on actual tracking conditions and theoretical analysis, the constraint conditions are given by Equation (20).

(20) $$\left\{\begin{array}{c} y\to y_{min}\\ 0.09\leq \varphi \leq 0.25\\ 3\leq v\leq 8\\ 1\leq d\leq 3 \end{array}\right.$$

The values of the optimal path tracking parameters were finally determined as 0.125 m^−1^, 3 km/h, and 1 m for curvature, speed, and spacing, respectively.

### 3.2. Tests in the Field

**Figure 11:**
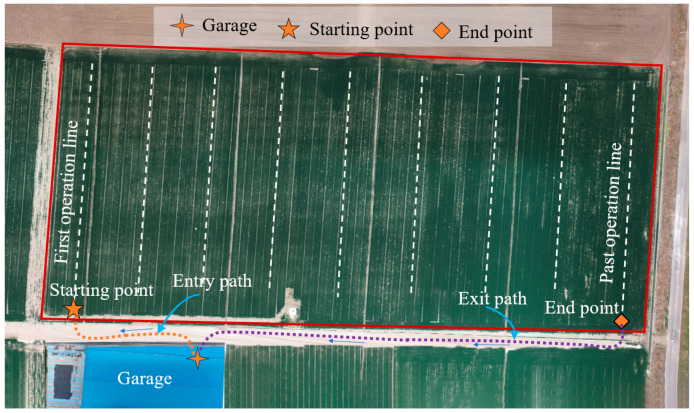
The test scene.

To evaluate the proposed ZPTM for the following zigzag paths, field tests were conducted using the optimal path tracking parameters to complete the automatic navigation process with the high-clearance sprayer. The test scene included a garage, field roads, and the working area, as shown in Figure 11. After departing from the garage, the high-clearance sprayer made a left turn and traveled along the entry path, then made a right turn to reach the starting point and entered the first working line. Once the field operations were completed, the high-clearance sprayer made a right turn at the end point of the last working line and continued straight along the exit path to return to the garage.

Considering the position of the working line and the optimal path tracking parameters, the entry and exit paths were planned by manually selecting anchor points in the orthophoto at an anchor point spacing of 1 m. The curvature at turns was set to 0.125 m^−1^, and the paths were stored in the point cloud as a navigation map. After the navigation terminal successfully loaded the navigation map, the high-clearance sprayer automatically tracked the entry and exit paths at a speed of 3 km/h and recorded the actual trajectory in real time.

**Figure 12:**
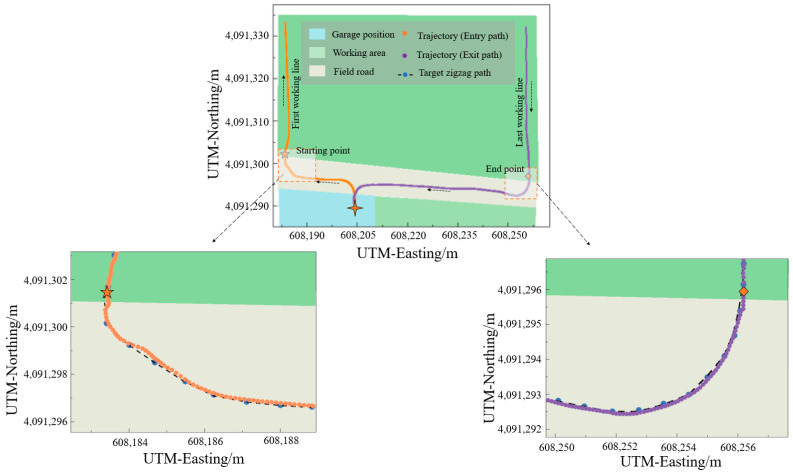
Actual trajectories and enlarged images of the high-clearance sprayer following the entry and exit paths.

During field tests, the entry and exit paths consisted of four turns and two straight lines. The turns at the starting and end points of field operations were particularly crucial in affecting the accuracy of field operations and the return to the garage. Therefore, the zigzag navigation process at the turns of the entry and exit paths was the focus of attention. Figure 12 shows the actual trajectories of the high-clearance sprayer following the entry and exit paths and the enlarged images of the turns at the starting and end points. For the yellow trajectory obtained along the entry path, the high-clearance sprayer could follow the entry path and reach the starting point. Due to the influence of the terrain potholes at the junction of the field boundary and road, the high-clearance sprayer oscillated during the turn process. However, it was able to quickly adjust and smoothly track the entry path to enter the first working line. For the purple trajectory obtained along the exit path, including the last working line of the field, the high-clearance sprayer successfully returned from the end point through the exit path to the garage.

Table 4 shows the lateral errors at the entry path, exit path, and turns at the starting and end points, respectively. For the turn at the starting point, the maximum, average, and RMS values of lateral errors were 3.3 cm, 1.86 cm, and 2.14 cm, respectively. For the turn at the end point, the maximum, average, and RMS values of lateral errors were 2.4 cm, 1.75 cm, and 1.98 cm, respectively. For the entry path, the maximum, average, and RMS values of lateral errors were 3.3 cm, 2.04 cm, and 2.27 cm, respectively. For the exit path, the maximum, average, and RMS values of lateral errors were 2.4 cm, 1.82 cm, and 2.06 cm, respectively. The maximum, average, and RMS values of lateral errors were almost the same for the entry path and the turn at the starting point. Similarly, the maximum, average, and RMS values of lateral errors were almost the same for the exit path and the turn at the end point. The maximum values of lateral errors for the entry and exit paths appeared at the turns of the starting and end points, respectively. This indicated that the turns of the starting and end points of the field had an important impact on the zigzag navigation accuracy. For the zigzag navigation, the maximum, average, and RMS values of lateral errors were 3.3 cm, 2.04 cm, and 2.27 cm, respectively. These results confirmed that the proposed ZPTM effectively planned zigzag paths and guided the agricultural vehicle to cover the working area with high accuracy and stability.

## 4. Conclusions

In this research, ZPTM was proposed as a method to describe a zigzag path using a point cloud consisting of anchor points with spatial information. These anchor points are obtained from orthophotos taken by UAVs and stored in a navigation map. The desired heading angle of the agricultural vehicle along the local target path, linked to adjacent anchor points, was calculated based on both lateral and heading errors to achieve highly precise zigzag path tracking. A GUI was designed on the navigation terminal to visualize the zigzag path tracking process of agricultural vehicles. The proposed ZPTM was applied on a high-clearance sprayer with automatic navigation, and zigzag navigation tests were conducted to explore the impact of path tracking parameters, including path curvature, moving speed, and spacing between anchor points, on navigation performance. The optimal path tracking parameters were then determined and adopted for field tests. The results indicated that the proposed ZPTM in this research was of adequate stability and applicability for zigzag navigation.

## Figures and Tables

**Table 1:** Technical parameters of the high-clearance sprayer.

Technical Parameter	Value
Motor power/kW	20
Tank volume/L	500
Wheelbase × Tread/m × m	1.5 × 1.5
Sprinkling width/m	12
Traving speed/km/h	0–10
Ground clearance/m	1.2
Minimum turn radius/m	3.5

**Table 2:** Path tracking errors for different navigation parameters.

Number	Curvature (m^−1^)	Speed (km/h)	Spacing (m)	Maximum (cm)	Average (cm)	RMS (cm)
1	0.09	3	1	3.7	1.42	1.37
2	0.09	4	1.5	3.3	1.32	1.29
3	0.09	5.5	2	3.1	1.46	2.67
4	0.09	7	2.5	4.3	2.02	2.39
5	0.09	8	3	4.8	2.24	2.52
6	0.11	4	1	4.2	1.37	1.21
7	0.11	8	1	3.2	1.33	1.86
8	0.11	3	1.5	3.6	1.43	1.55
9	0.11	7	2	4.4	1.76	2.11
10	0.11	5.5	2.5	3.5	1.64	1.65
11	0.11	4	3	4.4	1.84	2.22
12	0.125	3	1	2.1	0.84	0.85
13	0.125	7	1.5	3.9	1.53	1.18
14	0.125	8	1.5	2.8	1.23	1.82
15	0.125	5.5	2	2.2	0.94	1.45
16	0.125	4	2.5	3.9	1.57	1.78
17	0.125	5.5	3	3.3	1.84	2.04
18	0.17	7	1	4.3	2.02	1.79
19	0.17	5.5	1.5	3.7	1.67	1.98
20	0.17	4	2	5.2	1.69	1.87
21	0.17	8	2	3.5	1.43	0.99
22	0.17	3	2.5	3.7	1.76	2.39
23	0.17	7	3	5	2.24	2.36
24	0.25	3	1	4.1	1.73	2.25
25	0.25	5.5	1.5	6.8	2.24	2.11
26	0.25	7	2	4.2	1.72	2.72
27	0.25	8	2.5	6.3	2.49	2.33
28	0.25	4	3	6.5	2.65	2.72

**Table 3:** ANOVA results for regression model accuracy.

Source	Sum of Squares	df	Mean Square	F-Value	*p*-Value	
Model	4.40	4	1.10	8.06	0.0003	significant
*v*-Speed	0.8022	1	0.8022	5.88	0.0236	\
*d*-Spacing	1.68	1	1.68	12.31	0.0019	\
*φv*	0.7287	1	0.7287	5.34	0.0301	\
*φ* ^2^	1.38	1	1.38	10.10	0.0042	\

**Table 4:** Path tracking lateral errors.

Path	Maximum (cm)	Average (cm)	RMS (cm)
Turn at the starting point	3.30	1.86	2.14
Turn at the end point	2.40	1.75	1.98
Entry path	3.30	2.04	2.27
Exit path	2.40	1.82	2.06

## Data Availability

Data are contained within the article.
